# Supplementary material for: Altered microRNA Transcriptome in Cultured Human Liver Cells upon Infection with Ebola Virus
Source: Int J Mol Sci. 2021 Apr 6;22(7):3792. doi: 10.3390/ijms22073792 (PMC8038836; doi:10.3390/ijms22073792)
Supplement: Supplementary file 1 [file ijms-22-03792-s001.zip › Supplementary_File/C_ GO_Analysis_Results/16-30nt_go_Makona-96h-Huh7_vs_Control-96h-Huh7_down.mature_mirna_targets/MF_Pvalue_tree.pdf]

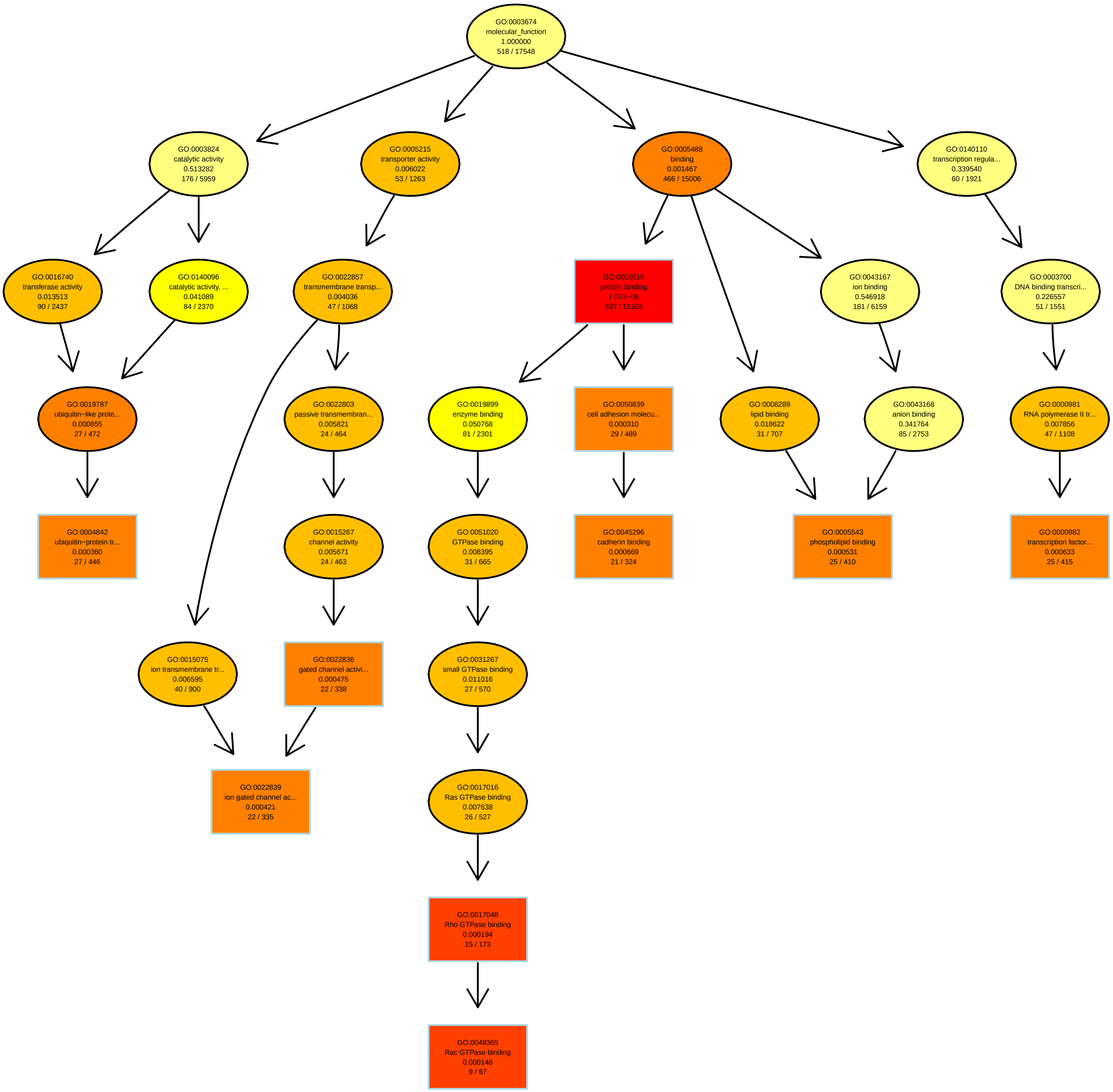
GO:0003674
molecular_function
1.000000
518 / 17548
GO:0003824
catalytic activity
0.513282
176 / 5959
GO:0005215
transporter activity
0.006022
53 / 1263
GO:0005488
binding
0.001467
466 / 15006
GO:0140110
transcription regula...
0.339540
60 / 1921
GO:0016740
transferase activity
0.013513
90 / 2437
GO:0140096
catalytic activity, ...
0.041089
84 / 2370
GO:0022857
transmembrane transp...
0.004036
47 / 1068
GO:0005515
protein binding
7.07e−06
387 / 11565
GO:0043167
ion binding
0.546918
181 / 6159
GO:0003700
DNA binding transcri...
0.226557
51 / 1551
GO:0019787
ubiquitin−like prote...
0.000855
27 / 472
GO:0022803
passive transmembran...
0.005821
24 / 464
GO:0019899
enzyme binding
0.050768
81 / 2301
GO:0050839
cell adhesion molecu...
0.000310
29 / 489
GO:0008289
lipid binding
0.018622
31 / 707
GO:0043168
anion binding
0.341764
85 / 2753
GO:0000981
RNA polymerase II tr...
0.007856
47 / 1108
GO:0004842
ubiquitin−protein tr...
0.000360
27 / 446
GO:0015267
channel activity
0.005671
24 / 463
GO:0051020
GTPase binding
0.008395
31 / 665
GO:0045296
cadherin binding
0.000669
21 / 324
GO:0005543
phospholipid binding
0.000531
25 / 410
GO:0000982
transcription factor...
0.000633
25 / 415
GO:0015075
ion transmembrane tr...
0.006595
40 / 900
GO:0022836
gated channel activi...
0.000475
22 / 338
GO:0031267
small GTPase binding
0.011016
27 / 570
GO:0022839
ion gated channel ac...
0.000421
22 / 335
GO:0017016
Ras GTPase binding
0.007638
26 / 527
GO:0017048
Rho GTPase binding
0.000194
15 / 173
GO:0048365
Rac GTPase binding
0.000148
9 / 67
